# Missing the Match Might Not Cost You the Game: Primer-Template Mismatches Studied in Different Hepatitis A Virus Variants

**DOI:** 10.1007/s12560-019-09387-z

**Published:** 2019-04-19

**Authors:** Sofia Persson, Måns Karlsson, Henrik Borsch-Reniers, Patrik Ellström, Ronnie Eriksson, Magnus Simonsson

**Affiliations:** 1 European Union Reference Laboratory (EURL) for Foodborne Viruses, National Food Agency, Hamnesplanaden 5, 453 23 Uppsala, Sweden; 2 Department of Medical Sciences, Zoonosis Science Centre, Uppsala University, Uppsala, Sweden; 3 Department of Mathematics, Stockholm University, Stockholm, Sweden; 4 Southern Roslagen Environmental and Health Authorities, Täby, Sweden

**Keywords:** Digital PCR, Real-time PCR, Reverse transcription, Primer, Mismatch, Hepatitis A virus

## Abstract

Mismatches between template sequences and reverse transcription (RT) or polymerase chain reaction (PCR) primers can lead to underestimation or false negative results during detection and quantification of sequence-diverse viruses. We performed an in silico inclusivity analysis of a widely used RT-PCR assay for detection of hepatitis A virus (HAV) in food, described in ISO 15216-1. One of the most common mismatches found was a single G (primer) to U (template) mismatch located at the terminal 3′-end of the reverse primer region. This mismatch was present in all genotype III sequences available in GenBank. Partial HAV genomes with common or potentially severe mismatches were produced by in vitro transcription and analysed using RT-ddPCR and RT-qPCR. When using standard conditions for RT-qPCR, the mismatch identified resulted in underestimation of the template concentration by a factor of 1.7–1.8 and an increase in 95% limit of detection from 8.6 to 19 copies/reaction. The effect of this mismatch was verified using full-length viral genomes. Here, the same mismatch resulted in underestimation of the template concentration by a factor of 2.8. For the partial genomes, the presence of additional mismatches resulted in underestimation of the template concentration by up to a factor of 232. Quantification by RT-ddPCR and RT-qPCR was equally affected during analysis of RNA templates with mismatches within the reverse primer region. However, on analysing DNA templates with the same mismatches, we found that ddPCR quantification was less affected by mismatches than qPCR due to the end-point detection technique.

## Introduction

Hepatitis A virus (HAV) is one of the leading causes of acute viral hepatitis worldwide (Murray et al. 2015). Transmission of HAV occurs mainly through the faecal–oral route by close person-to-person contact and via sewage-contaminated drinking water or contaminated food (Martin and Lemon 2006; Mast and Alter 1993; Murray et al. 2015). The virus belongs to the *Picornaviridae* family and the *Hepatovirus* genus, and the genome consists of a positive-sense, single-stranded RNA molecule of 7.5 kilobases, with a single open reading frame (ORF) flanked by 3′ and 5′ non-coding regions (NCRs) (Murray et al. 2015).

The human HAV strains are divided into three genotypes (I, II, III) and seven sub-genotypes (IA, IB, IC, IIA, IIB, IIIA and IIIB) (Pintó et al. 2012). Sub-genotypes IA and IB are the most abundant variants in most parts of the world, but sub-genotype IIIA is more common in south Asia (D’Andrea et al. 2015). However, strains of sub-genotype IIIA seem to emerge rapidly in other parts of the world (Mukomolov et al. 2012; Bosch et al. 2016; Miyamura et al. 2012) and the globalised food chain can be an important contributor to the spread of different HAV variants (Jacobsen 2018). In most cases, HAV infection do not cause any symptoms in individuals below 6 years of age, but strains of sub-genotype IIIA are more frequently associated with symptomatic infections in children (Miyamura et al. 2012; Bosch et al. 2016; D’Andrea et al. 2015). Strains of sub-genotype IIIA are also more often associated with fulminant hepatitis in adult patients compared with other sub-genotypes (D’Andrea et al. 2015; Bosch et al. 2016; Miyamura et al. 2012).

Recent outbreaks in the Nordic countries highlight the relevance of foodborne transmission of HAV (Nordic 2013; Enkirch et al. 2018; Rajiuddin et al. 2018). One-step reverse transcription quantitative real-time PCR (RT-qPCR) is the standard method for monitoring HAV contamination in the food chain. A single assay targeted within the 5′ NCR is often used for detection of genotypes I, II and III, and a new international standard was recently validated (Lowther et al. 2017) and published (ISO 2017) (ISO 15216-1, Microbiology of the food chain—Horizontal method for determination of hepatitis A virus and norovirus using real-time RT-PCR—Part 1: Method for quantification). The 5′ NCR is the most conserved part of the genome (Desbois et al. 2010), but even this region displays some degree of sequence heterogeneity. It is therefore almost impossible to completely avoid mismatches during RT-PCR assay design. Variation in template sequences, especially at or near the 3′ end of the primers, can decrease the efficiency of RT or PCR and thereby result in underestimation of the template concentration, false negative results and/or poor repeatability (Lefever et al. 2013; Stadhouders et al. 2010; Heitmann et al. 2005; Christopherson et al. 1997). Hepatitis A virus is often present in low numbers in food (Costafreda et al. 2006), and many techniques for concentration and extraction are inefficient and may lead to copurification of inhibitory substances for RT and/or PCR (Borgmastars et al. 2017; Bartsch et al. 2016; Schrader et al. 2012). These factors, in combination, make molecular detection of viruses in food and water particularly challenging (Mäde et al. 2013).

Droplet digital PCR (ddPCR) is increasingly being used as an alternative or complement to qPCR, mainly because it provides more precise quantification than qPCR (Persson et al. 2018; Hindson et al. 2013; Hayden et al. 2013). In contrast to qPCR, digital PCR relies on end-point detection, which makes it potentially less affected by sub-optimal PCR efficiency caused e.g. by inhibition (Coudray-Meunier et al. 2015; Racki et al. 2014) and primer-template mismatches (Strain et al. 2013; Hall Sedlak and Jerome 2014; Sedlak et al. 2017).

In this study, we performed an in silico inclusivity evaluation of the HAV RT-qPCR assay recommended by ISO 15216:1 (ISO 2017). The assay amplifies a 178 bp product at positions 68 to 241 (within the HAV reference genome NC001489). Four HAV sequences containing common or potentially severe mismatches were selected from GenBank for experimental analysis, and partial genomes were produced and analysed by both RT-ddPCR and RT-qPCR, to investigate in what degree quantification was affected. We also compared the effects of mismatches between RT-ddPCR and RT-qPCR. As contaminated foods often contain low levels of HAV (Costafreda et al. 2006), the sequence with one of the most common mismatch (a single G to U mismatch at the terminal 3′-end of the reverse primer region) was further analysed at low concentrations by RT-qPCR, to investigate how the probability of detection and limit of detection were affected. Lastly, clinical samples with sub-genotype IIIA, containing one of the most common mismatches, were analysed by RT-qPCR to investigate how the partial genomes used in the previous experiments reflect the behaviour of full-length viral genomes.

## Materials and Methods

### In Silico Inclusivity Analysis

HAV sequences were obtained from NCBI GenBank, using the query: “A”[porgn:__txid12092] NOT “A”[porgn:__txid12101] NOT modified[TITLE] NOT patent[TITLE]. FASTA files (complete records) were downloaded and genotyped with Hepatitis A Virus Genotyping Tool version 1.0 (National Institute for Public Health and the Environment, the Netherlands). Sequences belonging to genotypes other than I, II and III and sequences that could not be identified as HAV were removed, resulting in 7438 remaining sequences (as of 10 January 2018). Of these, 119 sequences contained the entire amplicon region of the investigated assay. The inclusivity of the assay was assessed by aligning the sequences with primer and probe sequences using CLC Main Workbench (Qiagen Bioinformatics, Aarhus, Denmark).

### Generation of HAV Sequences

Four sequences, designated A–D, with mismatches within the reverse primer region were selected for experimental investigation (Table 1). These are hereafter referred to as strains. Sequences from partial HAV genomes together with upstream T7-promoters and downstream HindIII sites were ligated into pEX-A2 vectors (Eurofins Genomics, Ebersberg, Germany). Plasmids were linearised with HindIII (New England Biolabs, Ipswich, United States) and separated by 1% agarose gel (Sigma-Aldrich, Saint Louis, United States) electrophoresis at 100 V for 2.5 h. Linear gel bands were excised and purified with QIAquick^®^ Gel Extraction Kit (Qiagen, Hilden, Germany). RNA transcripts were obtained using Riboprobe^®^ Combination System T7 RNA Polymerase (Promega, Madison, United States) and DNase-treated according to the manufacturer’s protocol. Clean-up of transcripts was carried out with an RNeasy MinElute Cleanup Kit (Qiagen). All transcripts were quality controlled by qPCR with and without RT, to ensure that the DNA content was below 0.1%. Quantification of plasmids and transcripts was conducted using a Qubit^®^ 3.0 Fluorometer together with the appropriate kit (Qubit^®^ dsDNA HS kit or Qubit^®^ RNA HS kit, depending on application) (Thermo Fisher Scientific, Waltham, United States). The material was diluted to appropriate concentrations in 1X TE buffer (Sigma-Aldrich, Saint Louis, United States), split into single-use aliquots and stored at − 70 °C.

**Table 1:** Template sequences used in this study

Sequence name	Nucleotide substitution(s) in the reverse primer region	GenBank accession no.	Genotype	Bases included in transcript (base number)
Quantification standard	None	NC001489	I.B	50-341
A	R: C19U	EU011791	III.A	24-272
B	R: U17C, C15U	AB839697	I.A	65-321
C	R: C19U, U18C	FJ360734	III.A	57-305
D	R: C19U, T18A, C15T	AB279734	III.A	30-321

R: reverse primer region, base 19 represents the position where the 3′-end of the primer binds. For instance, C19U means that a C is substituted to a U in the template at the position where the 19th base of the reverse primer binds

### Primers and Probes

Primers were purchased from Integrated DNA Technologies (San Jose, United States) and probes from Eurofins (Hilden, Germany). Perfectly matching (PM) primers were designed for each template sequence, to enable direct comparison between mismatching (MM) and PM primers (Table 2). As a change in one or a few bases can alter the thermal properties of the primers, and thereby potentially affect the efficiency of the RT-PCR reaction, the primer melting temperatures were evaluated using OligoAnalyzer 3.1 (Integrated DNA Technologies). The performance of the different primer systems was evaluated experimentally by analysing them in 10-fold dilution series with their respective PM templates using RT-qPCR. From this, PCR efficiencies, *y*-intercepts and determination coefficients were calculated. All five primer systems showed similar performance: PCR efficiency was between 95 and 100%, *y*-intercept was between 38.8 and 40.1 and coefficient of determination (*R*^2^ value) was between 0.99 and 1.00.

**Table 2:** Primers and probes used in this study

Type	Name	Sequence (5′–3′)^a^	*T*_*m*_ (°C)^b^	Reference
F	HAV68	TCACCGCCGTTTGCCTAG	59.1	Costafreda et al. (2006)
P	HAV150	[FAM]-CCTGAACCTGCAGGAATTAA-[MGB][EQ]	Not assessed	Costafreda et al. (2006)
R	HAV240	GGAGAGCCCTGGAAGAAAG	57.0	Costafreda et al. (2006)
R	PM A	GGAGAGCCCTGGAAGAAA*A*	56.4	This study
R	PM B	GGAGAGCCCTGGAA*A*A*G*AG	57.0	This study
R	PM C	GGAGAGCCCTGGAAGAA*GA*	58.1	This study
R	PM D	GGAGAGCCCTGGAA*A*AA*TA*	53.7	This study

*FAM* 6-carboxyfluorescein, *MGB* Minor Groove Binder, *EQ* eclipse quencher

^a^Differences from the original primers are displayed in italics

^b^Calculated by OligoAnalyzer 3.1 (Integrated DNA Technologies)

### One-Step RT-ddPCR

RT-ddPCR was performed with the One-Step RT-ddPCR Advanced Kit for Probes (Bio-Rad, Hercules, United States). The reaction mix contained 500 nM forward primer, 900 nM reverse primer and 100 nM probe. Each sample (5.5 µL sample and 16.5 µL reaction mix, i.e. 20 µL + 10%) was applied to a 96-well plate, with a final volume of 22 µL. A 20 µL aliquot was then transferred to DG8™ Cartridges (Bio-Rad), and 70 µL of Droplet Generation Oil for Probes were added (Bio-Rad). Droplets were generated with a QX200™ Droplet Generator (Bio-Rad). After droplet generation, 40 µL of droplet suspension (containing the entire 5 µL of sample and 15 µL of reaction mix) were transferred to a 96-well plate (Eppendorf, Hamburg, Germany). RT-PCR was performed in a T100™ Thermal Cycler (Bio-Rad) with RT at 50 °C for 1 h, reverse transcriptase inactivation and enzyme activation at 95 °C for 10 min, followed by 50 cycles of denaturation at 95 °C for 30 s, annealing/elongation at 60 °C for 1 min and a final enzyme deactivation step at 98 °C for 10 min. Plates were transferred to the QX200™ Droplet Digital™ PCR system (Bio-Rad) on either the same day or the day after the reaction. Results were visualised in QuantaSoft™ software version 1.7.4 (Bio-Rad). Thresholds were set manually using the fluorescence intensity of the no template controls (NTCs) within each run as reference.

### One-Step RT-qPCR

RT-qPCR was performed as recommended by ISO 15216-1, with an RNA UltraSense™ One-Step Quantitative RT-PCR System (Thermo Fisher Scientific) in LightCycler^®^ 480 Multiwell Plates on a LightCycler^®^ 96 System (both Roche, Basel, Switzerland). Each reaction contained 500 nM forward primer, 900 nM reverse primer and 250 nM probe. For each sample, a total of 25 µL reaction mix was prepared, with 20 µL of reagent and 5 µL of sample. RT-qPCR was run with RT at 55 °C for 1 h, followed by inactivation of the reverse transcriptase at 95 °C for 5 min. There were 45 cycles, with denaturation of the template at 95 °C for 15 s, annealing at 60 °C for 1 min and elongation at 65 °C for 1 min. The results were analysed with the LightCycler^®^ 96 software, version 1.1 (Roche). The Cq values were determined by the software. Quantification by RT-qPCR was performed using a tenfold dilution series of linearised plasmid DNA, ranging from 10^5^ to 10 copies/reaction (Table 1). Only one standard (Table 1) was used for quantification of the different primer combinations, as it became apparent that the different primer systems were similar in their performance (see section “Primers and Probes”).

### Primer-Template Mismatches in RT-ddPCR and RT-qPCR: Investigation of Effects on Quantification

All strains (A–D) were analysed by RT-qPCR and RT-ddPCR in concentrations of approximately 10^4^ copies/reaction. Both RNA and DNA templates were studied. At the time of the study, there was only one RT-kit available for one-step RT-ddPCR. As the two platforms had different kits and reaction conditions, the conditions described for RT-ddPCR (i.e. enzyme kit, volume, thermal profile, primer and probe concentrations) were evaluated on the qPCR platform as a control. In this way, each strain was investigated with three detection protocols: (i) RT-ddPCR, (ii) RT-qPCR as suggested by ISO 15216-1 (hereafter called RT-qPCR) and (iii) RT-qPCR with dd-kit. The strains were analysed with at least two replicate wells of MM primer and at least two replicate wells of PM primer. At least one well with no template control (NTC) was included for each mastermix. All quantities obtained by RT-PCR were log_10_-transformed, and the average log_10_ concentration of the replicate wells was used for subsequent calculation and statistical analysis. The entire process was repeated three times, resulting in four RT-PCR runs for each detection protocol, template and strain. For each sample, the log_10_ difference from PM was calculated by subtracting the measured concentration (on log_10_ scale) with the mean concentration of the corresponding PM samples (on log_10_ scale).

### Statistical Analysis to Measure Effects on Quantification

A linear additive mixed effects model was used to study how the effect of reverse primer mismatches (log_10_ differences from PM) varied between detection protocols (RT-ddPCR, RT-qPCR, RT-qPCR dd-kit) and nucleic acid types (DNA, RNA), and whether the outcome of different detection protocols depended on whether RNA or DNA template was used. The main interest was in studying how the effect of mismatches varies in general between the detection protocols. Hence, to increase the external validity of the results, strain was included as a random effect. All covariates were cross-sectional, meaning that no hierarchy was imposed on the model. Denoting the *j*th log_10_ difference by $Y_{j}$, $j = 1, \ldots ,96$, the corresponding detection protocol by $x_{1j}$, the corresponding nucleic acid type by $x_{2j}$ and the corresponding strain (A-D) by $z_{j}$, in formal notation the model (Model 1) becomes:

$$Y_{j} = \mu + \alpha_{{x_{1j} }} + \gamma_{{x_{2j} }} + \eta_{{x_{1j} x_{2j} }} + \delta_{{z_{j} }} + e_{j}$$

$$\delta_{zj} \sim N\left( {0,\sigma_{\delta }^{2} } \right)$$

$$e_{j} \sim N\left( {0,\sigma_{e}^{2} } \right)$$

where $\alpha$ is the effect of detection protocol, $\gamma$ is the effect of nucleic acid type, $\delta$ is the effect of strain, $\mu$ is the overall mean and $e_{j}$, $j = 1, \ldots ,96$ are the residuals and $\eta$ is the effect of interaction between detection protocol and nucleic acid type. The random effects of strain were assumed to come from a zero mean normal distribution with variance $\sigma_{\delta }^{2}$. The residual variance was assumed to be normal with mean zero and variance $\sigma_{\epsilon }^{2}$.

As we had a balanced design in our experiment the model was fitted using the *aov* function in R (R Development Core Team 2018). Pair-wise post hoc tests were performed to determine factor levels between which the differences were significant. For this, we used Tukey’s correction for multiple testing with the *emmeans* function from the *emmeans* package in R (Lenth 2018). An effect was considered significant at *p *≤ 0.05.

### A Primer-Template Mismatch at Low Template Concentrations: Effects on Limit of Detection

A two-fold dilution series was prepared with the RNA transcript for strain A (Table 1). Two RT-qPCR mastermixes were prepared, one containing PM primers and one containing MM primers. Eight dilution levels were included, and each dilution level was analysed in 16 replicate wells per mastermix. The RT-qPCR analysis was performed as described in ISO 15216-1 (ISO 2017)/Section “One-step RT-qPCR”.

### Statistical Analysis to Measure Effects on Limit of Detection

To determine whether there was a general difference between PM and MM primers in probability of detection $\pi_{i}$ across the dilution series, a multiple logistic regression model (Agresti 2003) was fitted with $\pi_{i}$ as a function of log_2_ expected concentration $c_{i}$ and primer type $P_{i}$. The expected concentrations were calculated by taking the geometric mean value of the concentrations measured in the PM wells at the undiluted level, followed by log_2_ transformation, and subtraction by one for each dilution level. In formal notation, the following model (Model 2) was fitted to the data:

$$logit\left( {\pi_{i} } \right) = \beta_{0} + \beta_{1} c_{i} + \beta_{2} P_{i}$$

$$D_{i} \sim Bernoulli\left( {\pi_{i} } \right)$$

where $D_{i} , i = 1, \ldots , 256$ is whether the sample was detected ($D_{i} = 1$) or not ($D_{i} = 0$). In this model, *P*_*i*_ was set to 0 if an MM primer was used and was set to 1 if a PM primer was used for sample well *i*. Thus, any effect of the primer type could be detected by the estimated value of $\beta_{2}$. The model was fitted using the *glm* function in R. An effect was considered significant if the *p* value of the parameter estimate was ≤ 0.05. The 95% limit of detection was defined as the lowest template concentration where the estimated probability of detection was 95% (Forootan et al. 2017). Hence, the 95% limits of detection for MM and PM primers could also be estimated by Model 2, by solving the linear predictor equation for *c* with π set to 0.95. A model allowing interaction effects between the two explanatory variables was also considered. Model fit was assessed by the Akaike information criterion (AIC) using the *aic* function in R.

### Verification of the Effect of a Common Mismatch Using Full-Length Viral Genomes

Four HAV positive human serum samples previously characterised as genotype IIIA, were kindly provided by the Public Health Agency of Sweden. Viral RNA was extracted with NucliSENS^®^ miniMAG^®^ (bioMérieaux, Marcy l’Etoile, France), according to the manufacturer’s instructions. Sample and elution volume was 200 µl. One-step RT-PCR was performed in a T100™ Thermal Cycler (Bio-Rad) using SuperScript III one-step RT-PCR system with platinum Taq DNA High Fidelity (Invitrogen, Carlsbad, United States). The assay amplified a region between base 34 to 463 (within NC001489) with 200 µM forward primer (CTCTTGGAAGTCCATGGTGAG) and 200 µM reverse primer (GCCGCTGTTACCCTATCCAA). The RT-PCR protocol was RT at 55 °C for 60 min, followed by inactivation of the reverse transcriptase/activation of the *Taq* polymerase at 94 °C and 45 cycles of 94 °C for 15 s, 60 °C for 1 min and 68 °C for 1 min. A final step occurred at 68 °C for 10 min. PCR products were purified using ExoSAP-IT™ PCR Product Cleanup Reagent (Thermo Fischer Scientific). Sanger sequencing was performed at Eurofins Genomics, using the same primers as above. To perform the experiment, the full-length viral genomes and the RNA transcript of strain A were diluted to a concentration of 100 copies/reaction. Two RT-qPCR mastermixes were prepared, one containing PM primers and one containing MM primers. Each sample was analysed in four replicate wells per mastermix. The RT-qPCR analysis was performed as described in ISO 15216-1 (ISO 2017)/Section “One-step RT-qPCR”. Two wells with no template control (NTC) were included for each mastermix. All quantities obtained by RT-qPCR were log_2_-transformed, and the average log_2_ concentration of the replicate wells was used for subsequent calculation and statistical analysis. For each well, the log_2_ difference from PM was calculated by subtracting the measured concentration (on log_2_ scale) with the mean concentration of the corresponding PM wells (on log_2_ scale).

### Statistical Analysis to Verify the Effect of a Mismatch

A hierarchical linear mixed effects model (Model 3) was used to study how the effect (log_2_ difference from PM) of the reverse primer mismatch represented by strain A varied depending on whether a partial genome (transcript) or a viral full-length genome (serum sample) was used as template. Four full-length genomes were included to increase the generality of the results. Viral sample was included as a random effect and nested within the fixed effect template type. In formal notation Model 3 becomes:

$$u_{k} = \mu + a_{{v_{{1_{k} }} }} + b_{{v_{{1_{k} }} v_{{2_{k} }} }} + \varepsilon_{k}$$

$$b_{{v_{{1_{k} }} v_{{2_{k} }} }} \sim N\left( {0,\sigma_{b}^{2} } \right)$$

$$\varepsilon_{k} \sim N\left( {0,\sigma_{e}^{2} } \right)$$

Where $\mu$ is the overall mean, *a* is the effect of template type and *b* is the effect of viral sample and $\varepsilon_{k} , k = 1, \ldots 20$ is the residual. The model was fitted using the *lmer* function from the *lme4* package (R Development Core Team 2018; Bates et al. 2014). The significance of the fixed effect in the model was assessed using an *F*-test. The effect was considered significant at *p *≤ 0.05.

## Results

### In Silico Inclusivity

Human HAV sequences containing the entire amplicon region (*n *= 119) were subjected to in silico inclusivity analysis (Table 3). Sub-genotype-wise multiple sequence alignments with the HAV-specific primers and probes recommended by ISO 15216-1 (ISO 2017) revealed that mismatches and/or insertions within the probe region were most common among genotype I, whereas mismatches at the 3′-end of the reverse primer region were most common among genotype III. All investigated sequences of genotype III displayed a mismatch at the position corresponding to the 3′-end of the reverse primer (a C to U substitution at position 223 within NC001489). Overall, 54% of the sequences investigated displayed at least one mismatch and/or insertion within the primer and/or probe regions.

**Table 3:** Summary of results of the in silico inclusivity analysis

Sub-genotype	Total number of sequences	Number of sequences with at least one mismatch/insertion	Within F region	Within P region	Within R region
I.A	54	10	1	10	3
I.B	36	28	0	26	2
II.A	1	1	0	0	1
II.B	1	0	0	0	0
III.A	16	16	1	2	16
III.B	4	4	0	0	4
Could not assign	7	0	0	0	0
All sequences	119	59	2	38	26

### Primer-Template Mismatches in RT-ddPCR and RT-qPCR: Effects on Quantification

**Fig. 1:**
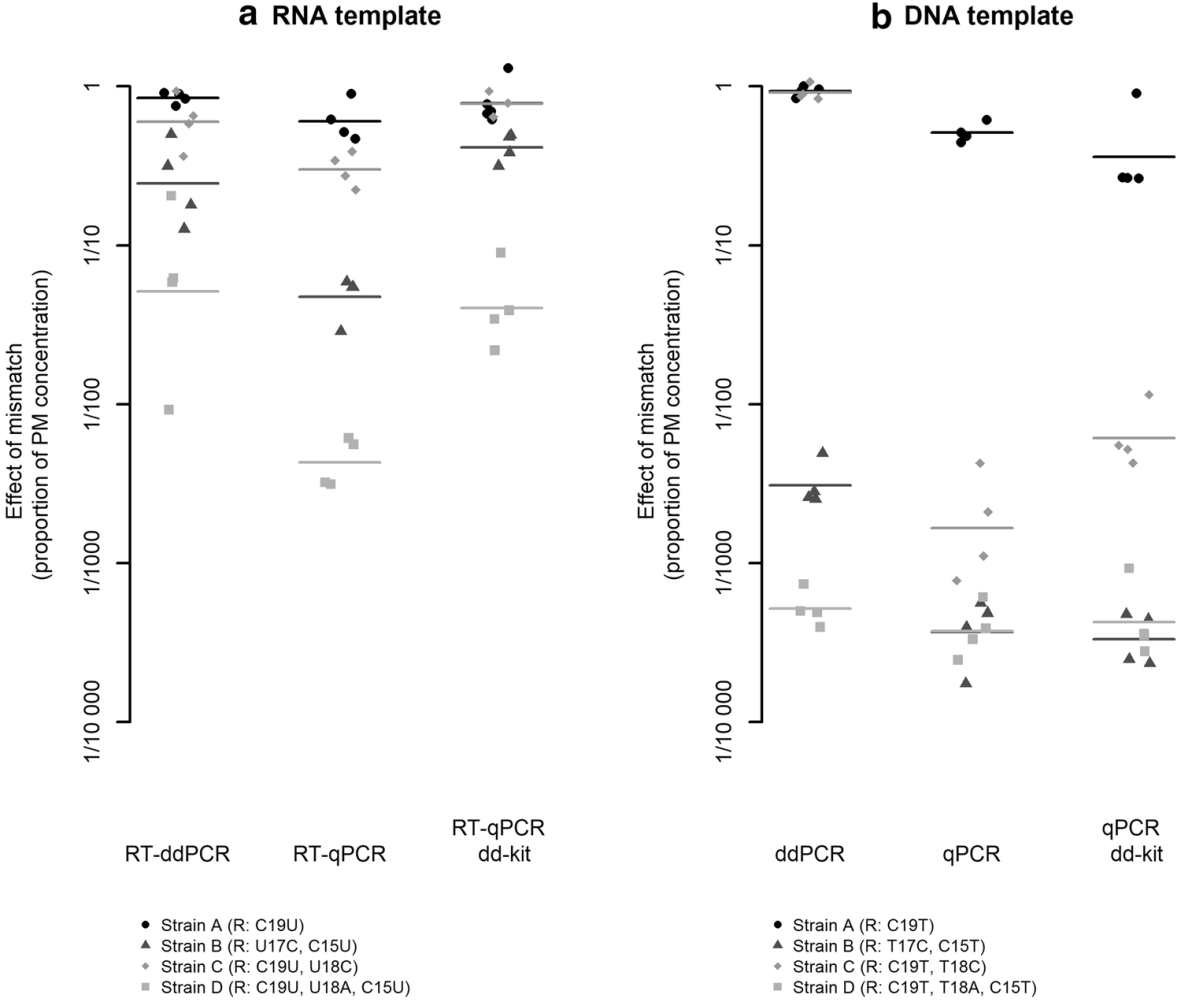
Mismatches within the reverse primer region. **a** Effects in one-step RT-PCR (RNA templates) and **b** effects in PCR (DNA templates). Four (RT)-PCR runs were conducted for each template and method, and each dot displays the average from a single run. The horizontal bars represent averages of four runs. R: reverse primer region. For instance, R: C19U means that a C is substituted to a U in the template at the position where the 19th base of the reverse primer binds. The 19^th^ base corresponds to the terminal 3′-end of the primer

Four sequences with common or potentially severe mismatches were analysed by RT-ddPCR and RT-qPCR (Table 1). All the mismatches investigated were located within the reverse primer region; forward primer and probe were perfectly matching. Strain A displayed one of the most common mismatching sequences, with a single G (primer) to U (template) mismatch at the terminal 3′end of the reverse primer. The differences in quantification between MM and PM for strain A were small (Fig. 1a). Using standard RT-qPCR conditions, the concentration of strain A was underestimated by a factor of 1.7 (− 0.22 on log_10_ scale). Strains B, C and D had two to three mismatches at and near the 3′ end of the reverse primer region. These variants were rare, with only one sequence of each present in the in silico inclusivity analysis. Strain D, with three mismatches at the 3′-end of the reverse primer region, was seemingly the most underestimated (Fig. 1a). Using standard RT-qPCR conditions, the concentration of strain D was underestimated by a factor of 232 (− 2.4 on log_10_ scale).

### Comparison Between RT-ddPCR and RT-qPCR

We studied how the quantitative effects of reverse primer mismatches varied between detection protocols (RT-ddPCR, RT-qPCR, RT-qPCR dd-kit). Both RNA (Fig. 1a) and DNA (Fig. 1b) templates were included, although of course the latter cannot reflect the real situation, as HAV is an RNA virus. From the parameter estimates of Model 1, we concluded that there was a significant (*p *= 0.011) interaction effect between detection protocol and nucleic acid type (i.e. whether RNA or DNA was used as template). Pair-wise post hoc tests revealed no significant differences between the three detection protocols for RNA templates, although there was a near-significant tendency for RT-qPCR to be more affected by mismatches than RT-ddPCR and RT-qPCR dd-kit (*p* = 0.092 and 0.090, respectively). No difference was observed between RT-ddPCR and RT-qPCR dd-kit (*p* = 1.0). For DNA templates, on the other hand, ddPCR quantification was less affected by mismatches than both qPCR and qPCR dd-kit (*p* < 0.01 in both cases), while no difference was observed between qPCR and qPCR dd-kit (*p* = 1.0). Furthermore, within each detection protocol, mismatching RNA templates were less underestimated than mismatching DNA templates (*p* < 0.01 for all detection protocols). The estimated effects are displayed in Table 4.

**Table 4:** Estimated effects of reverse primer mismatches (obtained from Model 1)

	RNA			DNA		
	RT-ddPCR	RT-qPCR	RT-qPCR, dd-kit	ddPCR	qPCR	qPCR, dd-kit
Degree of underestimation [log_10_ difference from PM (times underestimation on the linear scale)]	− 0.54 (3.5)	− 1.10 (12.6)	− 0.54 (3.5)	− 1.5 (29)	− 2.5 (295)	− 2.4 (229)

The value in each column describes the expected difference in concentration when using the MM under the setting given by the column headers, across the four strains tested

**Fig. 2:**
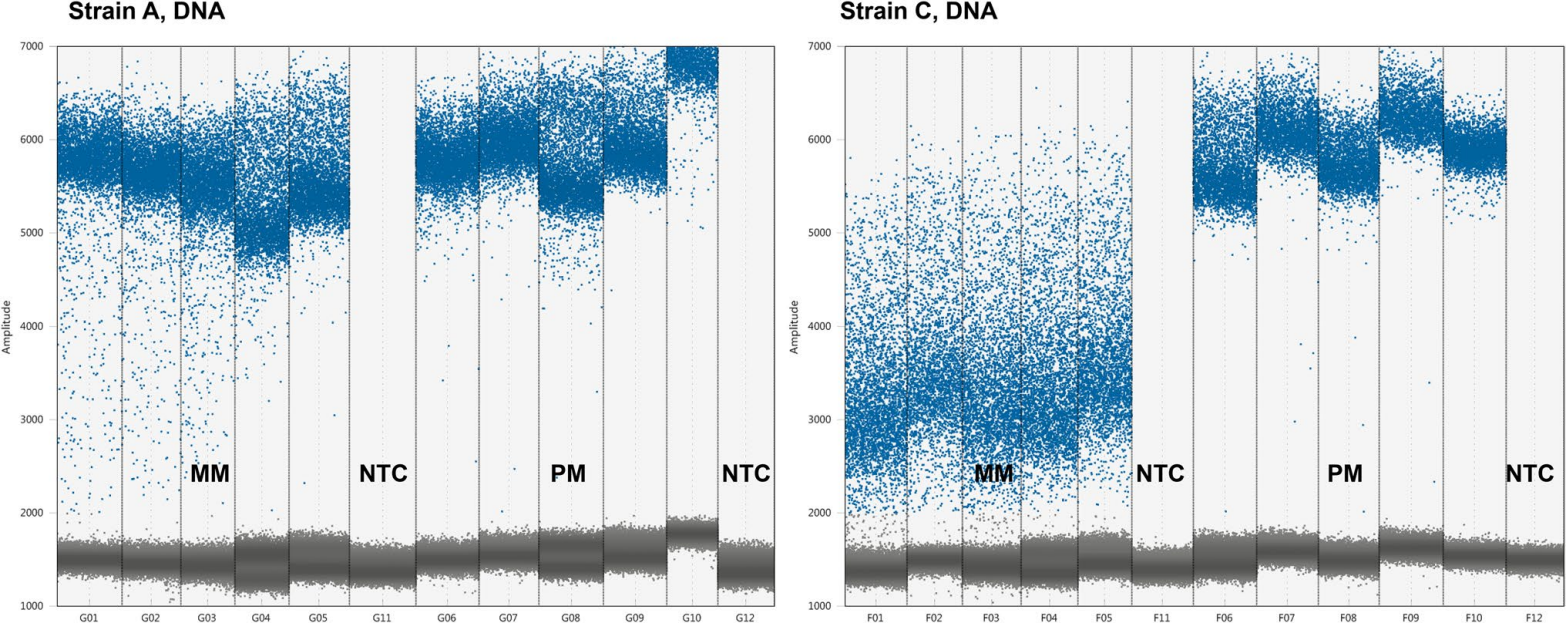
Mismatches and the performance of ddPCR. Fluorescence amplification plots of DNA templates for strain A and C in ddPCR, amplified with MM and PM primers, in a run containing five replicate wells of each sample. *NTC* no template control

The performance of ddPCR can be examined by looking at the separation between the positive and the negative droplet population and the proportion of ‘rain’ (droplets falling between the positive and negative droplet population) in the amplification plot. Poor separation and/or a high proportion of rain indicate impaired performance (Lievens et al. 2016). For the DNA templates of strain A and C, we observed that the ddPCR performance was impaired with MM primers as compared with PM primers (Fig. 2). This was not observed for RNA templates.

### A Common Primer-Template Mismatch at Low Template Concentrations: Effects on Limit of Detection

**Fig. 3:**
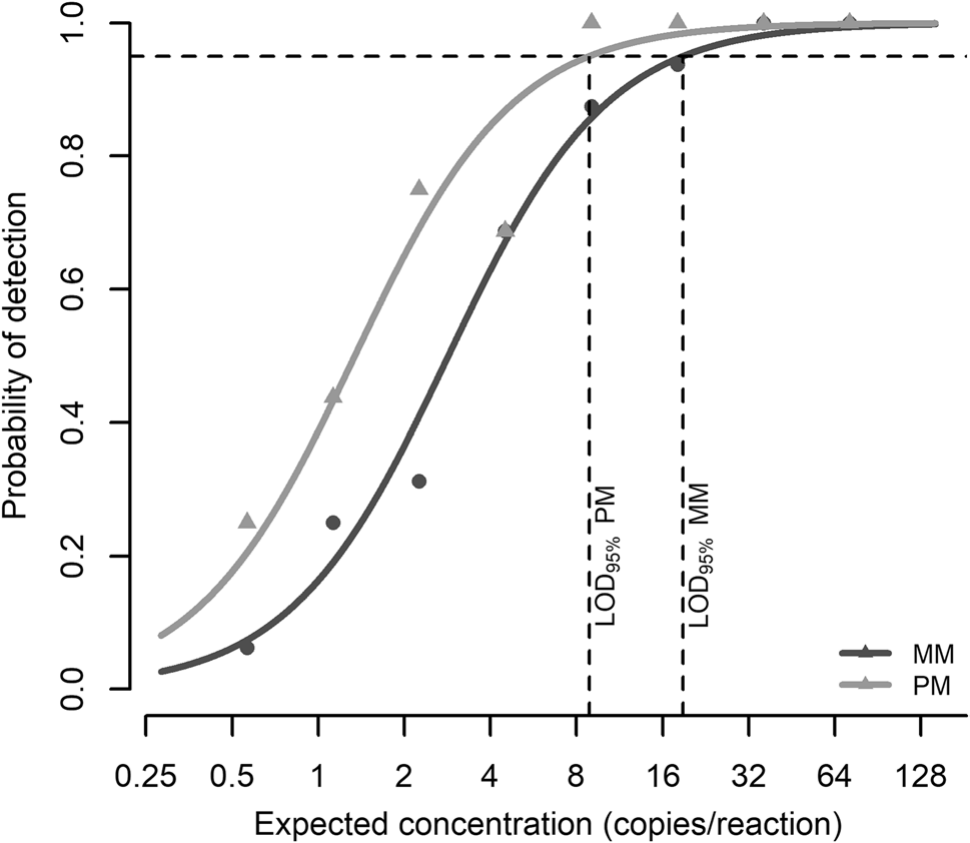
The RNA template of strain A in RT-qPCR at low concentrations. Strain A has a single G (primer) to U (template) mismatch at the terminal 3′-end of the reverse primer. The figure shows estimated probability of detection for MM and PM primers versus expected concentration. The observed proportions of positive samples are shown as dots and the estimated 95% limits of detection are indicated as vertical lines. *n *=16 tested sample wells per dilution level and primer type

The RNA template of strain A was further analysed at low concentrations by RT-qPCR. The estimate of parameter $\beta_{2}$ in Model 2 was 1.2 (on log_2_ scale), with an associated *p* value < 0.01, revealing that the probability of detection was lower when using MM primer than PM primer across the dilution levels tested (Fig. 3). The 95% limits of detection were 8.9 copies/reaction (3.1 log_2_ copies/reaction) with PM primer and 19 copies/reaction (4.2 log_2_ copies/reaction) with MM primer. Accordingly, the 95% limit of detection increased with a factor of 2.1 (1.1 on log_2_ scale) when using MM primer instead of PM primer. A model with an interaction term between primer type and dilution level was also evaluated (not shown), to study whether the effect of primer type was different at different dilution levels, but no significant interaction effect was found. This model did not provide better fit to the data than the model without the interaction term.

### A Common Primer-Template Mismatch: Verification Using Clinical Samples

**Fig. 4:**
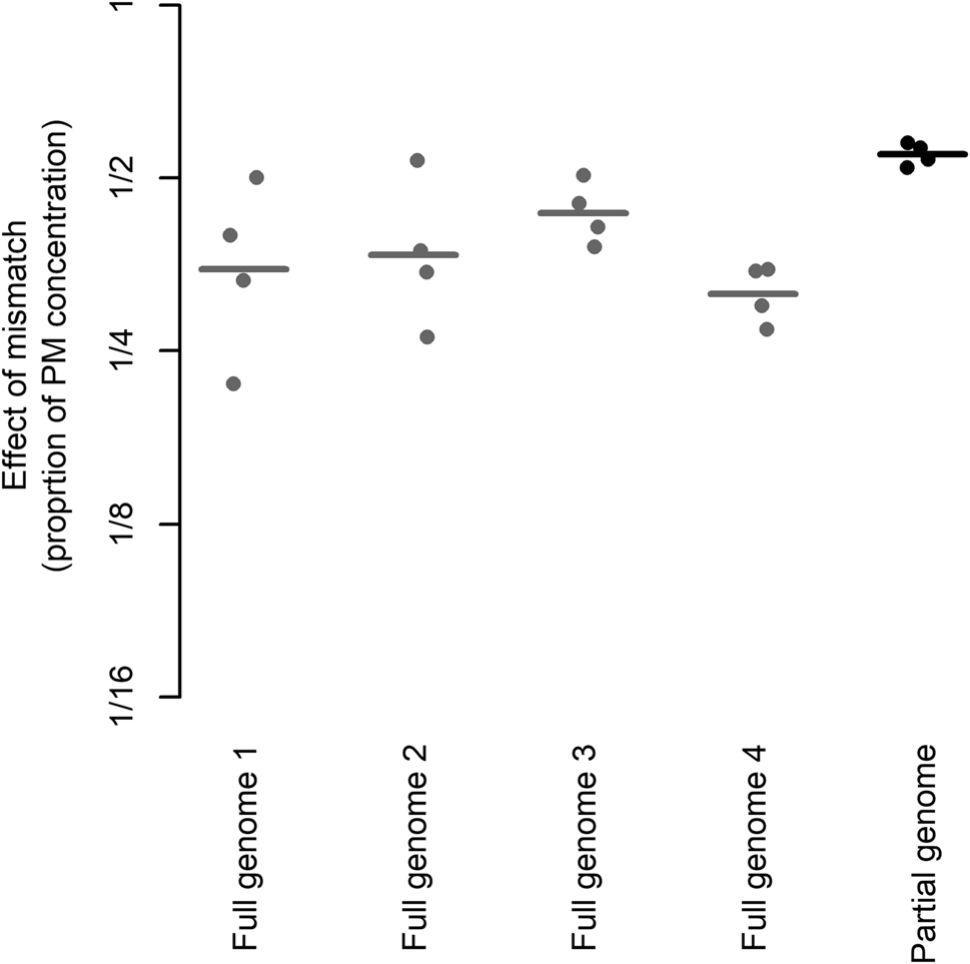
Partial genome of strain A and full-length genomes with the same mismatch in RT-qPCR. Dots represent single RT-qPCR wells and lines represent mean values

Four full-length HAV genomes characterised as genotype IIIA were collected. The sequence analysis revealed that all genomes contained exactly the same mismatch as strain A. The full-length genomes and the partial genome (the RNA transcript for strain A) were analysed using RT-qPCR (Fig. 4). By Model 3, it was found that full-length genomes were underestimated by a factor of 2.8 (− 1.5 on log_2_ scale) and the partial genome by a factor of 1.8 (− 0.86 on log_2_ scale). The *p* value associated with the parameter estimate for template type was 0.052. Furthermore, when comparing the PM and MM samples, we noticed a tendency towards larger variation in quantification between the wells for the MM samples of full-length genomes 1 and 2 as compared with their respective PM samples (data not shown).

## Discussion

More than half of the HAV sequences studied in silico displayed at least one mismatch and/or insertion with regard to the RT-PCR assay recommended by ISO 15216:1 (ISO 2017), which is a horizontal method widely used for quantitative detection of HAV in food. The fact that all genotype III sequences had at least one mismatch at the 3′-end of the reverse primer is worrying in terms of quantification accuracy and ultimately led us to perform this study. However, the experimental analysis revealed that quantification of the variant with a single mismatch at the terminal 3′-end of the reverse primer was affected to a relatively small extent (Figs. 1 and 4). Similar results have been reported previously (Stadhouders et al. 2010). In that study, single terminal 3′-end reverse primer mismatches generally had minor effects on quantification (less than 1 Cq increase) when using an M-MLV RT*/Taq* based RT-qPCR system (Stadhouders et al. 2010). Furthermore, the G to U base-pairing of strain A represents a purine to pyrimidine mismatch, which is known to have a lesser impact on (RT)-PCR quantification than pyrimidine to pyrimidine and purine to purine mismatches (Christopherson et al. 1997). The other variants (strains B–D) were rare. Therefore, we cannot rule out that these variants could be due to sequencing errors. Strain B belonged to genotype IA and was present in 1 of 54 genotype IA sequences found in GenBank. Strains C and D belonged to genotype IIIA. Each of them was present in 1 of 16 genotype IIIA sequences in GenBank.

At low concentrations, the mismatch of strain A caused an increase in 95% limit of detection by a factor of 2.1 when using the detection protocol recommended by ISO 15216-1 (Fig. 3). Although the effect was relatively small seen from an RT-PCR perspective, it is important to be aware of it, as outbreak-related food samples may contain very low levels of HAV. We investigated how the quantitative effects of mismatches varied between RT-ddPCR and RT-qPCR. Although no significant differences were observed for RNA templates, RT-qPCR was seemingly most affected by mismatches, while RT-ddPCR and RT-qPCR with dd-kit were equally affected, suggesting that the differences between the detection protocols were associated with enzyme kit and RT temperature, and not the method of detection (i.e. end-point versus real-time). In one-step RT-PCR-based detection of positive-sense single-stranded RNA viruses, the reverse primer is used to prime the RT step. In the first round of amplification of the cDNA template generated in the RT step, the forward primer binds the negative-sense cDNA, creating a positive-sense DNA sequence that perfectly matches the reverse primer in the next round of amplification. Hence, there are no additional effects of mismatches in the PCR step. The efficiency of the RT step is not considered during RT-ddPCR; instead, the number of amplifiable cDNA copies is quantified. This means that end-point detection will have no advantages over real-time detection when mismatches are present only in the reverse primer region. The main reason for the potential difference between the two kits is most likely the different temperatures used for RT; the RT-ddPCR kit uses 50 °C, whereas the RT-qPCR kit suggested by ISO 15216-1 uses 55 °C. Lower RT temperature allows for less specific hybridisation of DNA and RNA (Stadhouders et al. 2010), but too low temperatures may hamper RT of RNA molecules with strong secondary structures, by sterically hindering the primer binding or causing the reverse transcriptase to stall (Bustin et al. 2009; Bustin 2002). There is also an increased risk of the primer binding to non-target sequences at low temperatures. This may outcompete the desired reaction if the template of interest is present in very low concentrations (Bustin 2002).

The behaviour of the in vitro transcribed RNA templates used in this study may not completely represent the behaviour of full-length viral genomes. This issue was partially addressed in the last experiment, where full-length viral genomes containing the same mismatch as strain A were analysed together with the  corresponding in vitro transcribed RNA template (Fig. 4). Four full-length genomes were included to increase the generality of the results. By Model 3, we found a near-significant tendency that the full-length genomes were more underestimated than the partial genome, meaning that the effect of mismatches in full-length genomes may be larger than estimated in the experiments with partial genomes. However, the effect was small (Fig. 4), which means that the approach of using in vitro transcribed partial genomes can be an inexpensive and convenient alternative  for RT-PCR inclusivity testing when viral strains are not available. Unfortunately, we did not have access to full-length genomes containing the other mismatching variants (B–D) used in this study. The experiment was small, so we acknowledge the fact that there might be small differences between genomes with the same mismatch, and furthermore that these differences can be dependent on factors such as sequence variation outside the primer and amplicon regions inflicting different RNA secondary structures that in turn will be dependent on temperature and chemistry used in the RT step.

Two of the full-length genomes had noticeably larger SD between replicates when amplified with MM primer as compared with PM primer (data not shown). Increased variability in quantification due to mismatches has been observed previously (Lefever et al. 2013). A more complex RNA structure could potentially decrease accessibility for mismatch primers more than for perfect match primers.

We also investigated DNA templates and found that ddPCR quantification was less affected by mismatches due to the end-point detection technique. Although not relevant for molecular detection of HAV, this information is still valuable in a general context, as it reveals an advantage of ddPCR over qPCR. The performance characteristics of ddPCR, such as rain and separation between the positive and negative droplet population, were affected by mismatches. Impaired performance of a ddPCR assay may therefore be a potential warning sign for the occurrence of mismatches. Mismatches affect the amplification efficiency and thereby the fluorescence accumulation in both qPCR and ddPCR. In qPCR, this will result in an increase in Cq value and hence affect the quantification of the template. In ddPCR, this will result in droplets with lower fluorescence intensity (Bosman et al. 2015), which was clearly evident for e.g. strain C (Fig. 2). However, quantification will not be affected if these droplets remain above the threshold for a positive droplet. Although we did not compare the effects of mismatches of the different strains in separate statistical analyses, we observed that the DNA template of strain C displayed the greatest difference between ddPCR and qPCR (Fig. 1b). However, we do not understand why. It is clear that the effects of mismatches appear to be highly context-dependent (Stadhouders et al. 2010), and may thus be difficult to predict in silico. Experimental verification of the inclusivity is therefore necessary as a complement to in silico analyses (Hedman et al. 2018).

Lastly, we found that mismatching DNA templates were more underestimated than mismatching RNA templates. This is explained by the 5–10 °C lower temperature in the RT step compared with the annealing temperature during DNA amplification. Hence, under conditions similar to this study, mismatches in one-step RT-PCR will likely be less detrimental if located within the reverse primer region compared with the forward primer region.

Forward primer mismatches were not examined here, but two previous studies suggest that one-step RT-ddPCR underestimates viral RNA with forward primer mismatches to a lesser extent than one-step RT-qPCR (Sedlak et al. 2017; Strain et al. 2013). The effect of forward primer mismatches on RNA templates will arise during PCR amplification (the forward primer binds to cDNA), meaning that end-point detection will be an advantage in that case.

One limitation of this study was that the PM primers used to generate the reference quantities in some cases had different thermal characteristics than the original primers (Table 2). However, we evaluated the performance of the different RT-PCR-systems and concluded that the differences in primer characteristics had no major practical influence on the results.

Together, these results indicate that the HAV RT-PCR assay described in ISO 15216-1 (ISO 2017) underestimates the quantity of HAV genotype III sequences, and also that ddPCR quantification is more resilient to mismatches than qPCR, due to the end-point detection technique. Moreover, from a general point of view, it can be debated whether it is appropriate to use exactly the same set of primer and probe sequences for detection of sequence-variable viruses across many different laboratories and countries. The primer and probe sequences recommended by ISO 15216-1 (ISO 2017) are optional to use, but standardisation of RT-PCR assays may risk reducing the collective redundancy of detecting a broad range of sequence variants, and hence newly emerged variants are at risk of being undetected.
